# A new marseillevirus isolated in Southern Brazil from *Limnoperna fortunei*

**DOI:** 10.1038/srep35237

**Published:** 2016-10-14

**Authors:** Raíssa Nunes dos Santos, Fabrício Souza Campos, Nathalia Rammé Medeiros de Albuquerque, Fernando Finoketti, Rayra Almeida Côrrea, Lucia Cano-Ortiz, Felipe Lopes Assis, Thalita Souza Arantes, Paulo Michel Roehe, Ana Cláudia Franco

**Affiliations:** 1 Laboratório de Virologia, Departamento de Microbiologia, Imunologia e Parasitologia, Instituto de Ciências Básicas da Saúde (ICBS), Universidade Federal do Rio Grande do Sul (UFRGS), Avenida Sarmento Leite 500, SALA 315, Porto Alegre, CEP 90050-170, Rio Grande do Sul, Brasil; 2 Laboratório de Vírus, Departamento de Microbiologia, Instituto de Ciências Biológicas da UFMG, Avenida Presidente Antônio Carlos, 6627 Caixa Postal 486, Belo Horizonte, CEP 31270-901, Minas Gerais, Brasil

## Abstract

Members of the family *Marseilleviridae* are giant viruses that have the ability to infect amoebas. Such viruses were initially described in 2009. Since then, this family has grown, and diverse members have been found in different environments and geographic locations. Previous phylogenetic analyses suggested the existence of four marseillevirus lineages. A fourth lineage was described with the discovery of the *Brazilian marseillevirus* (BrMr), isolated from Pampulha Lake, Brazil. Here we describe the isolation and characterization of the *Golden marseillevirus* (GMar), a new marseillevirus isolated from golden mussels (*Limnoperna fortunei*) in South of Brazil. This new representative of *Marseilleviridae* has circular, double-stranded (dsDNA) that contains 360, 610 base pairs and encodes 483 open read frames (ORFs). The complete virus genome was sequenced and phylogenic analyses indicated clear differences between this virus and other marseilleviruses. In addition, this is the only marseillevirus so far that has been isolated from mussels, and this report expands the diversity of environments from which giant viruses could be recovered.

The discovery of protist-infecting giant viruses started with the isolation of *Acanthamoeba polyphaga mimivirus* (APMV) in 2003^1^. Following the discovery of mimivirus, a virus named *Marseillevirus* was isolated in 2007 on biofilm from a cooling tower near Paris^2^. In 2010, *Megavirus chilensis* was recovered from a seawater sample from the shores of Chile^3^. Soon after, in 2013, the largest of the giant viruses so far discovered, *Pandoravirus*, was recovered from Chilean shores and from a garden pond in Australia^4^. More recently, *Pithovirus sibericum*^5^ and *Mollivirus sibericum*^6^ were identified in thirty-thousand-year-old Siberian permafrost. These viruses exhibit astonishing features, with large capsids and genomes comparable in size to small bacteria^7^. The “cell-like” features in giant viruses have led numerous scientists to suggest fundamental concepts outside the report of these particular viruses. These features include the size of the virions and genomes and the observation that many genes have no evident homologs in other microorganisms and, hence, might come from still unknown sources. The principal theoretical developments proposed that the giant viruses are characterized by a “fourth domain of life” that is different from but comparable to the three cellular domains^8^. The discovery of these giant viruses has crossed some boundaries between viruses and cellular life^9^, although ribosomes remain a distinctive attribute of living microorganisms^10^. According to the Baltimore system, giant viruses are placed among other dsDNA viruses: the nucleocytoplasmic large-DNA viruses (NCLDVs). Although dsDNA viruses rarely appear to have a single evolutionary origin, the NCLDVs all contain five core genes and share 50 likely ancestral genes^10^. Initially, NCLDVs included four known families of large-DNA viruses infecting eukaryotes: *Poxyviridae*, *Asfaviridae*, *Iridoviridae*, and *Phycodnaviridae*. The list of NCLDVs increased with the discovery of mimiviruses^1^ and, more recently, marseilleviruses^2^,^11^.

The first member of *Marseilleviridae* was isolated from the water of a cooling tower in Paris in 2007^2^. Subsequently, another family member, *Lausannevirus*, was discovered in water samples collected from the river Seine in 2011^12^. In 2013, the *Cannes8* virus was discovered in southeastern France in water from a cooling tower^13^. *Tunisvirus* and *Fontaine Saint-Charles virus* were discovered in freshwater from decorative fountains in Tunisia and France, respectively^14^,^15^. The *Insectomime* virus was isolated from the internal organs and digestive tract of a dipteran larva known as the drone fly^16^. Recently, two other marseilleviruses were recovered: *Port-Miou* virus, isolated from a sample from a brackish submarine spring^17^, and the *Brazilian marseillevirus*, isolated from Pampulha Lake in Minas Gerais^18^.

As demonstrated in earlier studies, giant viruses may be isolated from diverse environments. Recently, using molecular and virological methods, one study has demonstrated that oysters are excellent sources for isolating mimiviruses, possibly due to their structure, which allows for the bioaccumulation of microorganisms like viruses and, probably, amoebas^19^. This has brought our attention to golden mussels (*Limnoperna fortunei*). These mollusks, which are native to the freshwater systems of China, Southeast Asia, are a mytilid invasive species of the Plata Basin, accidentally introduced in 1991 by ballast water^20^. After its introduction to the region, the geographical distribution of *L. fortunei* included Guaíba Lake at Rio Grande do Sul (southern Brazil), where it was first reported in 1998^21^.

In this manuscript, we demonstrate that mussels are possible sources for the isolation of marseilleviruses. The isolation of marseillevirus from mussels is important in order to increase the number of strategies for isolation using filter-feeding organisms. Here, we report the isolation, identification, and genomic analysis of the *Golden marseillevirus* (GMar), a new marseillevirus isolated from golden mussels collected in southern Brazil.

## Methods

### Study Area and Samples

**Figure 1:**
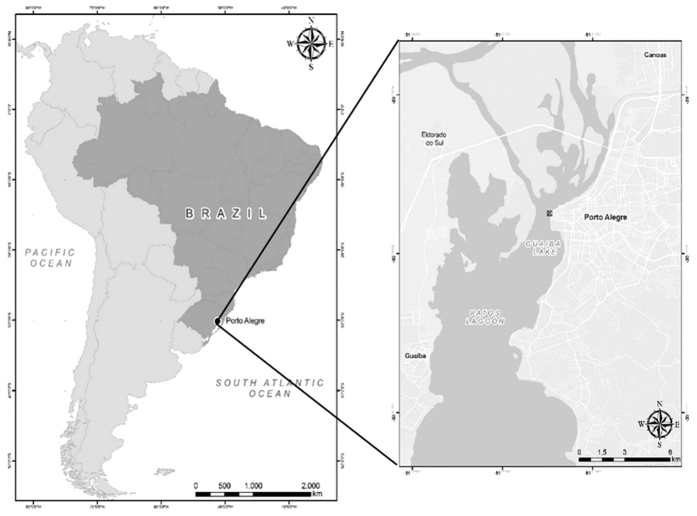
(**A**) Map of South America with emphasis on Brazil. (**B**) Region of Porto Alegre, capital of Rio Grande do Sul. The point of collection of the golden mussel specimens is marked with an [X]. (Data obtained from the IBGE database. Edited in QGis 2.10.1 http://www.qgis.org/).

Forty specimens of golden mussels (*L. fortunei*) were collected from Guaíba Lake, Rio Grande do Sul, Brazil, in July 2014 (30°01′59″S, 51°13′48″W; Fig. 1). The mussels were collected from a two meters deep location, and they were attached to a metal grid that had been submerged for six months before the date of collection. Due to the high human density and the concentration of industries around the lake, this region receives a large amount of domestic and industrial waste from three main rivers that form the lake’s origins: Gravataí, Sinos, and Caí rivers^18^.

### Preparation of samples for virus isolation

Forty golden mussels were submerged for 15 min in 70% ethanol for superficial shell decontamination. Subsequently, the valves were opened and the inner water was collected and diluted in 1 mL of saline buffer (PBS). The samples were pooled, totaling eight pools. These pools were homogenized with 1 mL of PBS, centrifuged at 10,000 × g, filtered through a 0.45-μm membrane and 10 U/μL of Penicillin-GIBCO^®^ by Life Technologies were added to the filtrate to avoid bacterial contamination.

### Isolation of GMar in free-living amoebas

Cells of *Acanthamoeba polyphaga* (genotype T4) were cultivated in 10 mL of Peptona-Yeast Extract-Glucose (PYG) medium at 30 °C in 25-cm^3^ culture flasks, supplemented with 50 μg of gentamicin. After 48 h, the cells were harvested and centrifuged. The pellet was re-suspended in sterile PAS (Page’s amoeba saline), and 10^4^ amoebas per well were cultured in 24-well microplates. After 24 h, 100 μL of the inoculum, prepared as mentioned above, were used to infect the *A. polyphaga* monolayers, which were then incubated for three days at 30 °C. This step was repeated by sub-culturing the primary culture onto fresh amoeba monolayers. Up to five blind passages were performed. The amoeba cells were assessed daily for the presence of viruses and for cytopathic effects on the cell such as lysis and cellular rounding. To evaluate the replication of GMar in different amoeba, monolayers of *Acanthamoeba castellani* was infected too.

### One-step growth curves

After the virus isolation, infectivity assays were performed by inoculating the *A. polyphaga* monolayers in the 24-well microplates with the virus isolate at an multiplicity of infection (MOI) of 10. The viruses were allowed to adsorb for 1 h, and then the cells were washed with PAS and incubated at 30 °C. At 1–14, 24, 25 and 26 h post-infection (pi), the cultures were frozen and thawed three times and then titrated. The virus titration was performed in 96-well microplates containing approximately 4 × 10^4^ *A. polyphaga* cells per well in 100 μL of the PYG medium. Virus titers were calculated according to the method of Spearmann and Kärber and expressed as the log_10_ culture infectious doses per 50 mL (TCID_50_/50 μL).

### Transmission electron microscopy

After the titrations, the *A. polyphaga* monolayers were infected at an MOI of 10. Uninfected cell cultures were used as controls. After 7 h of infection, the monolayers were washed twice with 0.1 M PBS and fixed with 2.5% glutaraldehyde (grade I) in 0.1 M PBS for 1 h at room temperature. Following the visualization of the full cytopathic effect (CPE), the cell monolayers were scraped off of the plates and pelleted by centrifugation at 900 × g for 5 min. The amoebae were then fixed with 2% osmium tetroxide and embedded in EPON resin. Ultrathin sections were stained with 2% uranyl acetate and examined using a Tecnai G2-Spirit FEI 2006 transmission electron microscope operating at 80 kV at the Microscopy Center, UFMG, Brazil.

### DNA extraction

The supernatants of the *A. polyphaga*-infected cells were collected, vigorously vortexed, and centrifuged at 5,000 × g for 5 min. The cell-free virus particles were pelleted on a 25% sucrose gradient by ultracentrifugation (Sorvall Combi) at 33,000 × g for 2 h at 4 °C. The pellet was re-suspended in Tris-EDTA-NaCl buffer (TEN). In order to remove the nucleic acids not protected by the capsid, the preparation was treated with 100 U of DNAse I (Roche) and 100 U of RNAse (Invitrogen) at 37 °C for 1 h. Next, the virus DNA was extracted using phenol-chloroform according to Sambrook and Russel^22^. The DNA was re-suspended in ultrapure water and the quality and amount of virus DNA was analyzed using a NanoSpec^®^ and Qubit apparatus (Life Technologies).

### Genome sequencing, assembly and annotation

Next-generation sequencing was performed in a MiSeq (Illumina) apparatus with pair-end applications (2 × 150 pb). The pair-end samples were prepared with a Nextera XT DNA sample prep kit. After sequencing, a total of 510,511 reads were *de novo* assembled using Geneious^®^ software. The transfer RNA (tRNA) sequences were analyzed by applying the tRNAscan-SE tool. Gene predictions were performed using FgenesV^23^ tool. Functional annotation was performed by BLAST searches against the UniProtkB using an e-threshold of 0.01 and by the set of clusters of orthologous groups of proteins (COGs) of the NCLDV (NCVCOGs). Finally, the genome annotation was manually revised. After assembly, the topology of the genome was determined *in silico* and by the amplification of specific genomic fragments (data not shown).

### Phylogenetic analyses and genomes comparison

Phylogenetic reconstruction based on individual and concatenated alignment of the five *Megavirales* core genes-namely the family B DNA polymerase, the D6/D11 helicase, the VV A18 helicase, the D5 primase-helicase, and major capsid protein. The amino acid sequences were aligned with Muscle software, and the phylogenetic tree was constructed in MEGA 7. We built a hierarchical-clustering based on the gene presence/absence patter of 5443 NCVOGs, using a MeV tool with Pearson correlation as distance metric. The average nucleotide identity using both best hits (one-way ANI) and reciprocal best hits (two-way ANI) between two genomic datasets as calculated.

## Results

### The isolation of GMar and the morphology of the infected cells

**Figure 2:**
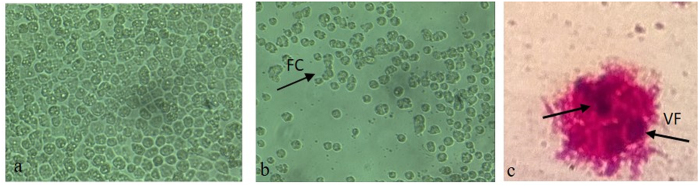
Cytopathic effect observed 10 h after the inoculation of *A. polyphaga* monolayers with the isolated virus (200×). Cells rounding, chain groups and lysis. (**a**) Negative control, uninfected amoeba culture; (**b**) Infected amoeba culture; FC: formation of chain groups.

The inoculation of *A. polyphaga* monolayers (Fig. 2a) resulted in three pools with clear cytopathic effects (ECP) showed in Fig. 2b. The CPE was characterized by the formation of cell chains, cell rounding, and lysis. In addition, the viruses were also inoculated in monolayers of *A. castellani* in order to identify the virus specificity. Following the infection, it was noticed that the viruses were able to replicate and cause cell lysis in both species of amoebas.

**Figure 3:**
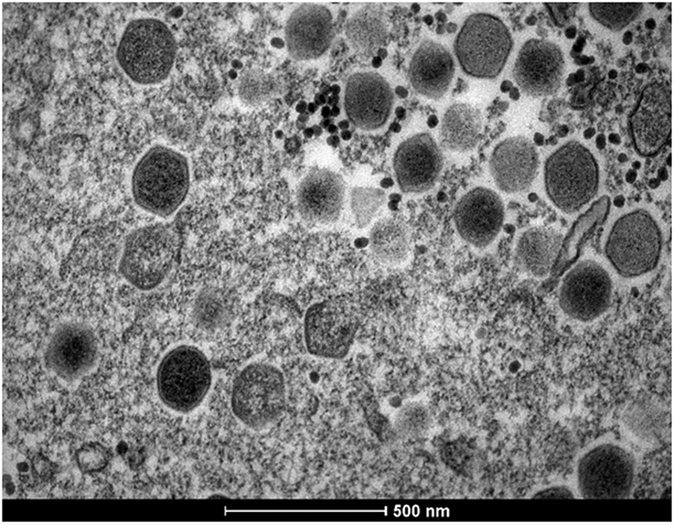
Transmission electron microscopy (TEM) image of *A. polyphaga* infected with the GMar. The image shows icosahedral particles inside an amoeba. A scale bar measured particles with 500 nm. We can observe different phases of viral morphogenesis.

For Transmission electron microscopy (TEM), a concentrated preparation of virus-infected *A. polyphaga* cells revealed markedly icosahedral particles of about 200 nm in diameter. The structure of the virus particles strongly resembled the structure of marseilleviruses (Fig. 3).

### One-step growth curve of GMar

**Figure 4:**
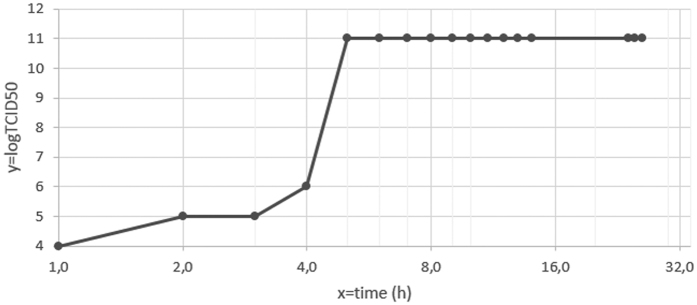
The infectivity assays were performed by inoculating *A. polyphaga* with the GMar at an MOI of 10. The viruses were allowed to adsorb for 1 h, and then the cells were washed with PBS and incubated at 30 °C. At 1–14 h and 24–26 h, the cultures were frozen and thawed three times and then titrated. A logarithmic curve expresses the titers measured at each time point pi. After 3 days of incubation, the titer (TCID_50_) was calculated as described by Reed and Muench^24^.

The replicative cycle of GMar was measured at different periods after infection. After 1 h pi, we observed cell alterations in the amoeba cytoplasm, described previously like as cell rounding. At this time, high virus titers (10^4^/50 μL) were detected. After 5 h pi, high virus titers were detected, as shown in Fig. 4 (10^11^/50 μL). At this time, we observed cell rounding and cell chains formation. After 10 h pi, it was possible to visualize extensive cell lysis, showed in Fig. 2 and the high titers were maintained. Figure 4 shows an exponential increase of infectious viral particles within 3–5 h pi. The Fig. 4 shows the replicative cycle of GMar. It is interesting to note that the cycle was completed in less than 24 h. After 1 h, we could detect virus and levels increased exponentially from the 3 h of infection. The highest titles were observed after 5 h.

### GMar genome features

The complete genome of three isolates that induced CPE in *A. polyphaga* was fully sequenced with a 217 coverage. The nucleotide sequences of the other two viruses were identical to the first one, and consequently, they were considered clones (data not show). The virus genome of one isolate (GenBank submission KT835053) is a circular, double-stranded DNA molecule composed of 360, 610 bp, which is comparable in size to the genomes, described previously, which range from 346, 754 bp to 386, 631 bp for *Lausannevirus* and *Insectomime* virus respectively. The mean guanine-cytosine is 43.1% for GMar, which is also similar to other members of this family.

A total of 483 open reading frames (ORFs) were identified after merging all coding sequence predictions. These ORFs were evenly distributed on both negative (263 ORFs) and positive (220 ORFs) strands, similar to the gene distribution on the *Brazilian marseillevirus* genome (261 and 230 ORFs on negative and positive strands, respectively). The GMar virus gene content consists of 48.03% uncharacterized proteins (232 of the 483 predicted proteins). The predicted proteins contained between 35 and 1293 amino acids. Moreover, we found numerous paralogous families, consisting of genes that code endonucleases (seven sequences), serine/threonine protein kinases (sixteen sequences), F-box containing protein (three sequences), putative MutH/archeal (four sequences) and ankyrin repeat (ten sequences). In addition, we found two histone-like proteins: a histone H2B/H2A fusion protein and a histone H3 containing a N-terminal Histone-like transcription factor, an archeal histone domain and C-terminal H3-like domain (252, 844- > 252, 089 and 252, 549- > 253, 574). The histone-like proteins in GMar resemble those predicted in other marseilleviruses, such as *Lausannevirus*^12^ and *Brazilian marseillevirus*^18^.

A total of 440/483 ORFs (91%) from the GMar had a significant BLASTp matches to marseilleviruses protein sequences available in the NCBI non redundant database. The 43 ORFs that displayed no identity with previously described sequences were tentatively classified as ORFans. As for the other marseilleviruses, no tRNA-related genes were detected in the GMar genome.

### Comparative genome

**Figure 5:**
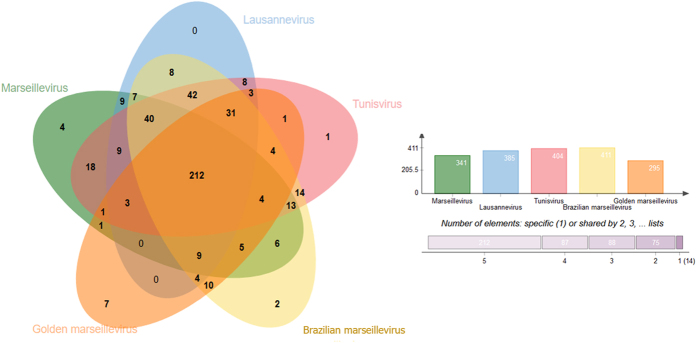
Distribution of orthologous genes clusters among marseilleviruses from one representative of each lineage (**A**: *Marseillevirus*; **B**: *Lausannevirus*; **C**: *Tunisvirus* and **D**: *Brazilian marseillevirus*) and GMar. The graph shows the number of orthologous genes that were used in this analysis.

Furthermore, we noticed a high proportion of orthologous genes (Fig. 5) in members of *Marseilleviridae*. Figure 5 shows that 212 orthologous genes are shared by *Marseillevirus* (Lineage A), *Lausannevirus* (Lineage B), *Tunisvirus* (Lineage C), *Brazilian marseillevirus* (Lineage D) and *Golden marseillevirus*. It is interesting to note that there are fourteen (14) non-shared genes, among these there are seven (7) GMar genes.

### Phylogeny

**Figure 6:**
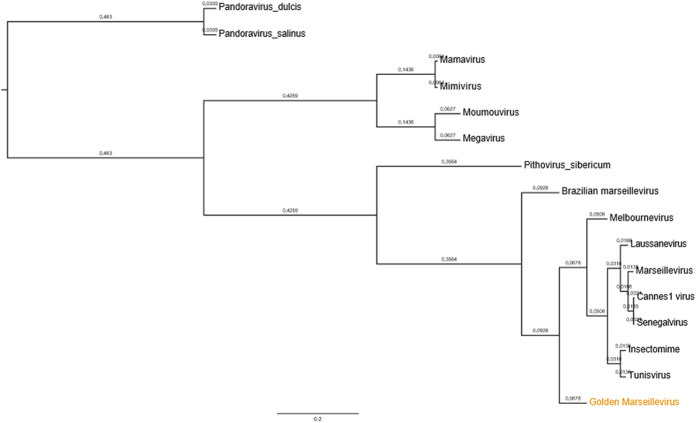
Hierarchical clustering tree based on phyletic patterns. Phylogeny based on presence/absence matrix of 5443 NCVOG (clusters of orthologous genes shared by NCLDVs). The Pearson correlation was used as metric distance, and the scale bar means the branch time.

A hierarchical clustering tree was constructed using presence/absence matrix of 5443 clusters of orthologous genes shared by NCLDVs. It shows in Fig. 6 that GMar is distant from other known lineages proposed, including the lineage D, recently discovered.

**Figure 7:**
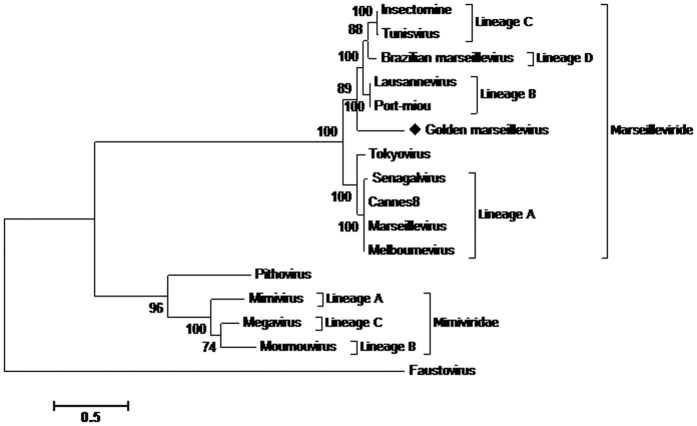
Phylogenetic reconstruction based on a concatenated alignment of the five core genes, DNA polymerase **B**, major capsid protein, VV-A18 helicase, D6/D11 helicase and D5 helicase. The amino acid sequences were aligned using Muscle. Evolutionary history was inferred by using the Maximum Likelihood method based on the JTT matrix-based model. The analysis involved 16 amino acid sequences. All positions containing gaps and missing data were eliminated. There were a total of 2552 positions in the final dataset. Evolutionary analyses were conducted in MEGA7^25^.

Phylogenetic analysis based on the core genes (DNA polymerase B, the VV A18-helicase, the D5 helicase, the D6/D11 helicase and the major capsid protein), for concatenated alignment (Fig. 7) show four clades for the previously described lineages, with a first group consisting of lineage A (*Marseillevirus*, *Senegalvirus*, *Melbournevirus* and *Cannes*8 virus). Three other clades appear to delimit the phylogeny of the *Marseilleviridae* family, composing the lineages previously known as B, C and D and a fifth comprising of GMar. These trees show the GMar within the family *Marseilleviridae*, but far from the other members already described. The analysis of nucleotide identity between the BrMr, recently founder of a new lineage and geographically closer to GMar, displays 74.96% of similarity. In addition to the concatenated tree, five trees with separate genes were also generated to support this result, based on the sequences available in the database. In spite of some variations in the positions, the clusters formed were the same, reinforcing the proposition of a new lineage to the virus here described (Supplementary data).

## Discussion

The discovery of a new strain of marseillevirus was attained by analyzing golden mussels, invasive organisms that are found worldwide. The source of the GMar is the inner water collected from golden mussels found in a local lake in Porto Alegre, Brazil, and this is the first report on the use of this organism for the isolation of marseilleviruses. Although this is a small-scale study, our results suggest that the golden mussels seem appropriate for the recovery of GMar and, perhaps, other marseilleviruses. It is likely that the filtration process necessary for the ingestion of nutrients of these organisms may be responsible for the accumulation of such viruses in their tissues. Oysters have been previously shown to be excellent sources for the isolation of mimiviruses due to their highly efficient filtration capacity, filtering more than 400 liters of water per day^19^. From the findings reported here, it seems that golden mussels behave similarly. By concentrating the water contaminants, the species was a rich source of giant viruses. In the near future, evaluations of golden mussels as accumulator of other giant viruses should be pursued.

Two members of the *Marseilleviridae* already described (*Marseillevirus* and *Insectomime*) can replicate in *A. castellani*, even if they have been isolated in *A. polyphaga*^2^,^16^. Our isolate share the same characteristic, as cytopathic effects is observed detected in both species. When comparing the replication profile of GMar with other marseilleviruses we observed additional similarities. After 3 h pi a MET of GMar infected amoebas revealed large viral factories which occupied half of the amoeba cytoplasm^26^. All virus factories contained different stages of the virion morphogenesis. At four hours pi we detected high titers of virus and one hour after that, an exponential increase in virus titers was observed.

In this study, we propose the foundation of a new lineage (E) in the *Marseilleviridae* family, of which GMar would be the first member. This suggestion is validated by genome analyses emphasizing several divergences between GMar and other marseilleviruses. When we look at the shared orthologues genes, we observe that seven genes are unique to GMar. It is noteworthy that the core genome analysis clearly distinguishes GMar from other lineages, drawing attention to the similar conserved gene content of the presently known marseilleviruses. More remarkably, phylogeny based on gene presence/absence patterns NCVOG reflects the gene loss and gain history of the giant viruses, clustering GMar into a distinctive clade in *Marseilleviridae* family. Conclusively, phylogenetic analysis show the four lineages of *Marseilleviridae*, which are presently composed by A, B, C and D. Recently a marseillevirus isolated in a river in Tokyo^27^ had its genome sequenced, however phylogenetic analyses are still missing to determine to which lineage this virus belongs. In our analyses, we included this new marseillevirus, and one can see that it’s in a new branch on the tree next to the lineage A. Based on concatenated alignment of five core genes, GMar probably founds a fifth clade in the marseilleviruses phylogeny. Even though the virus here described displays notable differences when compared to the other members of the *Marseilleviridae*, phylogenetic analysis shows that it is distant to *Mimiviridae* and the other giant viruses already identified. The analysis of separate genes also reinforces and consistently maintains the GMar in a separate branch in tree, indicating the possibility of a new strain. Finally, phylogenetic analysis of both the concatenated genes how the individual genes (according to availability in the database), shows clearly the four strains already described and characterized the GMar in one branch distant.

As the knowledge about the giant viruses is something recent, and the database grows with each new study, it is obvious that very soon there will be a review of annotation of genomes available, and probably a reorganization of the family *Marseilleviridae* with the inclusion of new members. In brief, these data increase the knowledge about the biological and genomic characteristic of marseilleviruses, currently incomplete.

## Additional Information

**How to cite this article**: dos Santos, R. N. *et al*. A new marseillevirus isolated in Southern Brazil from *Limnoperna fortunei*. *Sci. Rep*. **6**, 35237; doi: 10.1038/srep35237 (2016).

## Supplementary Material

Supplementary Information
