# Supplementary material for: A new marseillevirus isolated in Southern Brazil from Limnoperna fortunei
Source: Sci Rep. 2016 Oct 14;6:35237. doi: 10.1038/srep35237 (PMC5064363; doi:10.1038/srep35237)
Supplement: Supplementary Information [file srep35237-s1.docx]

**Supplementary Information**

**A new marseillevirus isolated in Southern Brazil from *Limnoperna fortunei***

Raíssa Nunes dos Santos¹, Fabrício Souza Campos¹, Nathalia Rammé Medeiros de Albuquerque¹, Fernando Finoketti¹, Rayra Almeida Côrrea¹, Lucia Cano Ortiz¹, Felipe Lopes Assis², Thalita Souza Arantes², Paulo Michel Roehe¹, Ana Cláudia Franco¹.

¹ Laboratório de Virologia, Departamento de Microbiologia, Imunologia e Parasitologia, Instituto de Ciências Básicas da Saúde (ICBS), Universidade Federal do Rio Grande do Sul (UFRGS). Avenida Sarmento Leite 500, SALA 315, Porto Alegre, CEP 90050-170, Rio Grande do Sul, Brasil.

² Laboratório de Vírus, Departamento de Microbiologia, Instituto de Ciências Biológicas da UFMG, Avenida Presidente Antônio Carlos, 6627 Caixa Postal 486, Belo Horizonte, CEP 31270-901, Minas Gerais, Brasil.

*Corresponding author: Santos, R.N., Laboratório de Virologia, Departamento de Microbiologia, Imunologia e Parasitologia, Instituto de Ciências Básicas da Saúde (ICBS), Universidade Federal do Rio Grande do Sul (UFRGS). Avenida Sarmento Leite 500, Porto Alegre, CEP 90050-170, Rio Grande do Sul, Brasil. +555133083655

E-mail engraissanunes@gmail.com

Phylogenetic reconstruction based on a alignment of the five core genes individually: DNA polymerase B, major capsid protein, VV-A18 helicase, D6/D11 helicase and D5 helicase. The amino acid sequences were aligned using Muscle. Evolutionary history was inferred by using the Maximum Likelihood method based on the JTT matrix-based model.

**FIGURE A)** DNA polymerase B

**Figure B)** VV-A18 helicase

**Figure C)** Major capsid protein

**Figure D)** D5-HELICASE

**Figure E)** D6/D11 HELICASE
